# The “great live and move challenge”: a program to promote physical activity among children aged 7–11 years. Design and implementation of a cluster-randomized controlled trial

**DOI:** 10.1186/s12889-019-6648-x

**Published:** 2019-04-03

**Authors:** Florence Cousson-Gélie, Marion Carayol, Bruno Fregeac, Lucile Mora, Florian Jeanleboeuf, Olivier Coste, Bruno Pereira, Mathieu Gourlan

**Affiliations:** 1 University Paul Valéry Montpellier 3, University Montpellier, EPSYLON EA 4556, F34000 Montpellier, France; 2 Epidaure, Prevention Department of the Institut régional du Cancer de Montpellier (ICM), Parc Euromédecine, 208 Avenue des Apothicaires, 34298 Montpellier cedex 5, France; 3 Academic resource center of Hérault dedicated to health promotion, 208 Avenue des Apothicaires, 34298 Montpellier cedex 5, France; 4 Direction Régionale Jeunesse Sport et Cohésion Sociale Occitanie, 3, avenue Charles Flahault, 34094 Montpellier Cedex 5, France; 5 Clermont-Ferrand University Hospital, Biostatistics Unit (Délégation Recherche Clinique et Innovation), Villa annexe IFSI, 58 rue Montalembert, 63003 Clermont Ferrand, France

**Keywords:** Physical activity, Randomized controlled trial, Theory of planned behavior, Primary prevention, Public health, Primary school

## Abstract

**Background:**

Recent population-based surveys have reported that large majorities of children in France, Europe and in the US are not complying with international physical activity (PA) guidelines. There is, therefore, a need to find programs that will improve children’s PA habits from an early age. Theory-based interventions that include school, family, and community involvement have the potential to generate a considerable increase in the PA level of children. The theory of planned behavior (TPB) is one of the most widely tested models of the factors influencing health-related behaviors. The Great Live and Move Challenge (GLMC) is an extended TPB-based intervention designed to promote PA in French primary school children aged 7–11 years. The objective of this paper is to describe the protocol of a randomized controlled trial to evaluate the effectiveness of the GLMC on the PA level of children.

**Methods:**

This is a two-year cluster-randomized controlled trial comparing an intervention group to a control group, randomized into clusters (community of communes) and stratified by department (Hérault, Gard, Aude) and residential environment (urban, rural). The goal is to recruit 4000 children. The GLMC involves children and their parents, and multiple local grassroots partners, such as school teachers, municipal officials and policy stakeholders. The intervention will be delivered over 3.5 months per year for a two-year period. Pre- and post-intervention, children and parents will be asked to fulfill a questionnaire concerning current PA level, TPB variables (i.e.*,* intentions, attitudes, subjective norms, perceived behavioral control) and other psychosocial variables (e.g.*,* perceptions of activity opportunities). A subsample of 400 children will be proposed to wear an accelerometer (i.e.*,* the Actigraph GT3X+). The primary hypothesis is that the GLMC intervention will increase the proportion of children achieving the World Health Organization’s recommended 60 min of moderate to vigorous PA per day by 15%.

**Discussion:**

This study will evaluate the effectiveness of a multilevel, theory-based PA program and potentially provide valuable information for schools and public health officers looking for innovative PA programs.

**Trial registration:**

ISRCTN:61116221, 19/06/2018.

## Background

There is ample evidence that being physically active is a key protecting factor against non-communicable diseases that develop over the lifespan (e.g., coronary heart disease, cancer) [1–3]. International guidelines recommend a minimum of 60 min of moderate to vigorous physical activity (PA) per day for children [4, 5], but recent population-based surveys have reported globally insufficient PA levels in young people: more than 80% of US adolescents [6], 66% of European adolescents [4], and 69% of school-aged children in France were not complying with international guidelines [7]. Childhood (i.e., 4–12 years old) is considered a critical period for the formation of PA habits [8], notably in the perspective of preventing PA decrease reported during the adolescence (i.e., 12–19 years old) [9, 10]. Hence the promotion of PA in school-aged children is a public health priority of the World Health Organization [11].

Recent meta-analyses of controlled trials of interventions intended to promote PA in children have revealed that they produce significant increases in PA level, but the effects are small [12] (Cohen’s *d* was 0.07 in children less than 10 years old) and the quality of the evidence is limited [12, 13]. Finding out how to enhance the effectiveness of PA programs in children is therefore a big challenge. Recent evidence suggests that multilevel interventions involving school, family and community are the best way of increasing PA [14]. Involving schools has the advantage that all children, including those from the most socioeconomically deprived communities, are exposed to the intervention. In addition, involving parents is important in promoting parental commitment to ensuring children undertake regular PA. Thus the involvement of public policy stakeholders appears essential to influencing the physical environment in which children and their family live [14–16].

Most of previous interventions have suffered from failure to understand the psychosocial mechanisms underlying behavioral change [17–19]. Implementing interventions based on a psychosocial theory is one way to identify the principle psychosocial variables related to target behaviors, thus enabling the selection of relevant intervention techniques [20]. The theory of planned behavior (TPB) [21] is one of the most widely tested models of the factors influencing health-related behaviors [22] and has been shown to be particularly suited to prediction of PA [23].

The Great Live & Move Challenge (GLMC) is a multilevel, extended TPB-based program which aims to promote PA amongst school-aged children (7–11 years old). This paper describes the protocol of a cluster-randomized controlled trial evaluating the effectiveness of the GLMC. Three factors make the GLMC study an original contribution to research on promotion of PA in children: (i) the use of a theory-based approach that follows current recommendations about linking behavioral change techniques to the theoretically derived target variables [24], (ii) the integration of 3 levels of PA promotion (i.e., school, family, and community) and (iii) the inclusion of an objective method of measuring of the impact of the intervention on PA (i.e., accelerometers), which remains rare in studies of children [12].

## Methods/design

The protocol was approved by the French Advisory Committee on Information-Processing in Material Research in the Field of Health (Comité Consultatif sur le Traitement de l’Information en matière de Recherche dans le domaine de la Santé, CCTIRS) (registration no. 15279) and the French Data Protection Authority (Commission Nationale de l’Informatique et des Libertés, CNIL) (registration no. 1860542). Any modifications to the protocol will be agreed by the sponsor (INCa) and ethic committee prior to implementation and notified to the health authorities in accordance with local regulations.

### Study objectives

**Fig. 1:**
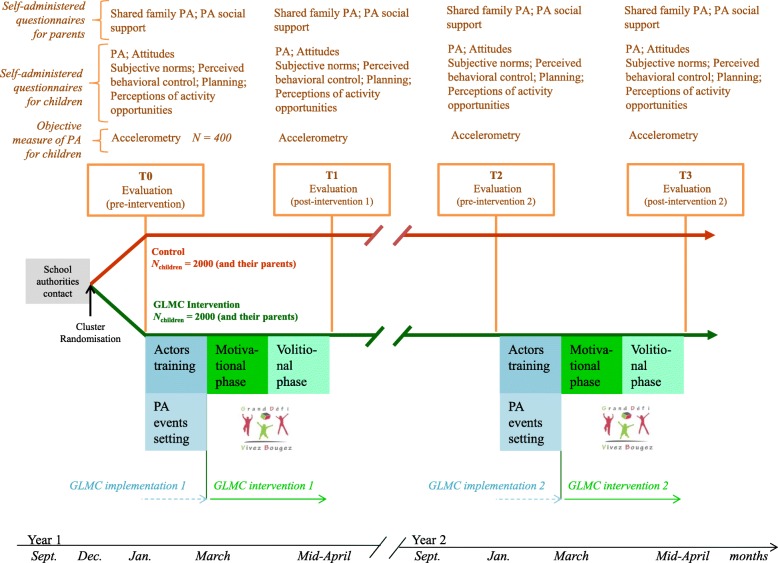
Design of the Great Live and Move Challenge cluster randomized controlled trial. *Note*: The number of expected children in both groups has been estimated by a power calculation. Specific to cluster-randomized controlled trial design

The primary objective of this study is to measure the effects of the multilevel extended TPB-based GLMC intervention on PA; the intervention is intended to increase by 15% the proportion of children meeting the current international recommendation of 60 min of moderate to vigorous PA per day at 24-month follow-up (i.e.*,* T3-post-intervention 2; see Fig. 1)*.*

The secondary objectives are to measure the effects of the GLMC on: *(i)* children’s scores for intentions, attitudes, subjective norms, perceived behavioral control, PA planning and level of perceptions of activity opportunities; and *(ii)* parents’ social support for their children’s PA and parental involvement in shared family PA.

### Study design and population

All the year 3 and year 4 children attending primary schools in the Hérault, Gard, and Aude departments of France (and their parents) are eligible to participate in the study. There is wide variation in the sociodemographic background and PA levels of children attending schools in these departments. The intervention involves children and their parents, and is implemented by multiple local, grass-roots partners, namely, school teachers, municipal officials and policy stakeholders from community agencies and district councils. To avoid contamination between the intervention and control groups randomization will be done at community of communes level (a community of communes is an administrative grouping of several cities or villages), thus all the schools in a given community of communes will represent a cluster.

The study is a two-armed prospective cluster-randomized, controlled intervention trial. Communities of communes will be randomized in equal proportions to one of the two study arms: 1) the GLMC experimental arm, which will receive a 24-month (6 weeks per year) multilevel, extended TPB-based program intended to promote PA; 2) the control arm, which will not receive any intervention (Fig. 1).

**Fig. 2:**
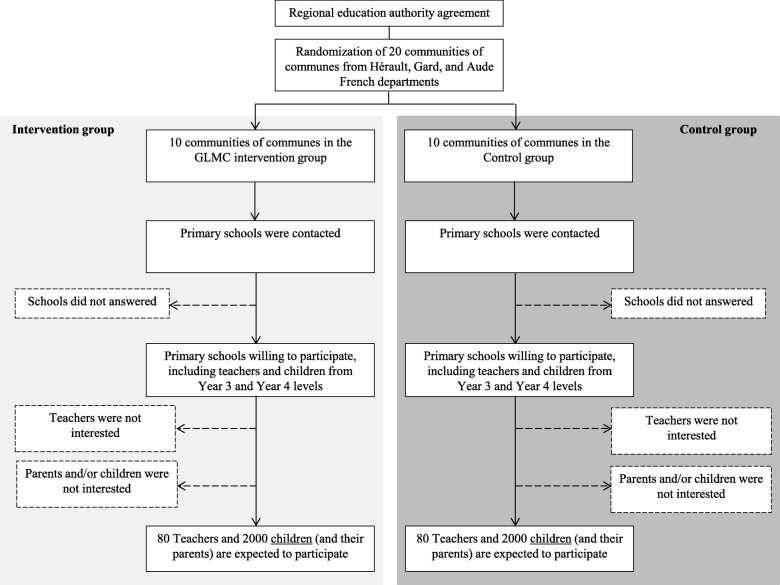
Planned flow diagram

After randomization, schools belonging to the communities of communes and their teachers will be informed about the study and their arm allocation by a research assistant. A member of staff outside the research team will introduce data into the computer in separate datasheets so that the researchers can analyze data without having access to information about the allocation. Eligible children from schools and classes willing to participate in the study, and their parents, will receive a written note explaining the research goal, design, assessment protocol (including information about the wearing of accelerometers) and intervention content (see flow diagram of schedule in Fig. 2). Children with medical conditions or and children taking medication that would limit their physical activity will not be included. Children will be given consent forms to take home for their parents to sign. There are three independent consent forms providing: *(i)* consent to the child filling in questionnaires; *(ii)* consent to fill in the parental questionnaires; *(iii)* consent to the child wearing an accelerometer.

### Randomization

The cluster randomization of communities of communes will computer-generated, using minimization and stratification, by an independent statistician (DRCI University Hospital of Clermont-Ferrand, France), using the Stata software (StataCorp, College Station, Texas, US). The randomization will be stratified by department and residential environment (urban; rural) to avoid unbalanced representation of communities of communes with respect to these criteria.

### Intervention

#### Rationale and theoretical framework: applying and extending the TPB model

**Fig. 3:**
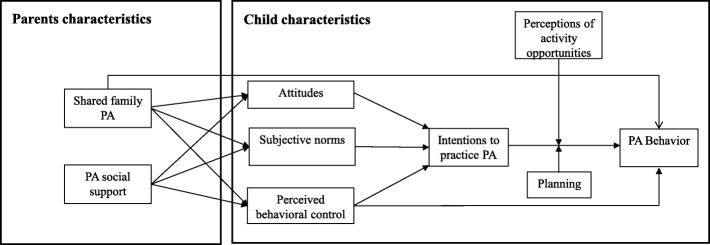
The “extended” theory of planned behavior model of the Great Live and Move Challenge. *Note*: PA = physical activity

The GLMC is a multilevel intervention carried out in school, family and community settings. The multilevel framework targets both personal and environmental factors that are important to behavioral change [25], particularly in children [26]. The TPB model appears a particularly suitable foundation for the GLMC intervention as it includes intrapersonal (e.g.*,* attitudes) and environmental variables (e.g.*,* subjective norms) [21]. According to Ajzen [21], intentions are the proximal determinant of behavior and reflect one’s motivation to perform a given behavior. Intentions are determined by three factors: attitudes, subjective norms and perceived behavioral control. Attitudes can be defined as the overall positive or negative evaluation of the target behavior and has both an affective (e.g.*,* enjoyable vs. unenjoyable) and an instrumental (e.g.*,* beneficial vs. harmful) component. Subjective norms also consist of two related components: a descriptive norm is an individual’s perception of how often important others (e.g.*,* friends, siblings) display a given behavior; whereas an injunctive norm represents an individual’s perception of how much others want him/her to perform a given behavior. Finally, perceived behavioral control is one’s perception of one’s ability to perform a given behavior in line with intentions. It is hypothesized that perceived behavioral control directly predicts performance of the relevant behavior (Fig. 3).

The TPB has been used extensively to study a broad range of behaviors [27], particularly health behaviors [22, 23], including PA in adults [28], children [29–31] and adolescents [32]. Meta-analyses of cross-sectional [33] and longitudinal [23] studies have provided evidence that TPB variables can explain PA behavior. The TPB’s capacity to predict intentions from attitudes, subjective norms and perceived behavioral control has been validated [34], however inconsistencies in the relationship between intentions and PA behavior have been reported. First, a recent meta-analysis of experimental studies revealed that intentions had only a very small (*d* = 0.15; 95% confidence interval = 0.06; 0.23) effect on PA behavior [35]. Second, longitudinal studies have shown that only 54% of participants translate their positive intentions into actual PA [36]. This discordance between intended and actual PA behavior is referred to as the ‘intention-PA gap’, and numerous authors have called for the investigation of factors that could reduce the gap and enhance the TPB’s predictive power [37]. A potential explanation for the gap is that there are mechanisms which moderate the relationship between intentions and behavior [38, 39]. Numerous scholars have called for exploration of extensions to the TPB that could plug the intentions-behavior gap and enhance the explanatory power of the TPB [40]. It is worth noting that recent research has shown that the intentions-PA relationship is moderated by planning [41] and activity opportunities [30]. Planning can be defined as a self-regulatory strategy for when, where and how one will perform a given behavior [42]. Opportunities for activity can be defined as the daily opportunities a child has for PA [43]. Based on this research we decided to base the GLMC intervention for children on an extended version of the original TPB including planning and perceptions of activity opportunities as moderators of the relationship between intentions and PA as well as the original TPB variables (attitudes; subjective norms; perceived behavioral control; intentions) (Fig. 3). As parental support for children’s PA (expressed as encouragement, pride and contingent feedback) and parental involvement in shared family PA are recognized as key parental behavioral factors promoting an active lifestyle adoption in children [16, 44], these variables were also included in the theoretical model underlying the GLMC (Fig. 3).

#### Design and implementation

##### Overview

The intervention is applied to children and their parents and involves multiple local grassroots partners, such as school teachers and municipal officials, as well as policy stakeholders from town councils and communities of communes. The GLMC will be delivered over three and a half months per year (from January to mid-April), for two years. It comprises two main steps: (1) The first two months are devoted to the implementation of the GLMC with local partners; (2) The last month and a half is devoted to a two-phase intervention for parents and children: (2*a*) a two-week ‘motivational’ phase intended to increase intentions to perform PA; (2*b*) a one-month ‘volitional’ phase intended to facilitate the translation of increased intentions into an active lifestyle [40] (Fig. 1).

Throughout the intervention, the Montpellier Cancer Institute (MCI)-Epidaure staff will coordinate the collaboration of the various actors and provide assistance, or even conduct sessions when necessary, as well as being available to answer questions at all times.

### Step 1: GLMC implementation with local partners

#### Training of teachers and municipal officials

Teachers and municipal officials will deliver GLMC sessions in their school and recreation center, respectively. Teachers are involved in both the motivational and volitional phases of the GLMC intervention. Their training consists of a six-hour educational course validated by the French Department of Education and they receive a pedagogical guide specifically designed for them. The course is organized in five parts covering *(i)* the structure and staff of MCI-Epidaure, *(ii)* the concept of PA (definition; impact of PA on wellbeing), *(iii)* the general principles of the GLMC (e.g.*,* the ‘energy cubes’, the collaborative nature of the intervention and the roles of teachers, parents and local policy stakeholders), *(iv)* the GLMC as a theory-based intervention (introduction to the TPB; explanation of the link between the theoretical variables and the sessions of the GLMC) and *(v)* the teacher’s guide to the GMLC.

Municipal officials are involved in delivering the volitional phase of the GLMC via sessions in schools and recreation centers. They receive a three-hour training course consisting of educational course and specially designed practical guide. The training is in three parts and covers *(i)* the concept of PA, *(ii)* the general principles of the GLMC, *(iii)* the municipal official’s practical guide to the GMLC.

#### Preparation with local policy stakeholders

Town councils and community of communes will be involved in the implementation and promotion of the GLMC in daily places and environment of families (e.g.*,* municipal squares, parks). They are involved in the volitional phase of the GLMC intervention (see below). All these local policy stakeholders are similarly involved in *(i)* planning ‘PA events’ (e.g.*,* family hikes), *(ii)* organizing the promotion of these events (e.g.*,* advertising in papers, websites and posters). The nature and characteristics of the PA events will be determined by local policy stakeholders. The MCI-Epidaure staff are in charge of advising and helping all entities with delivery of PA events. Specifically, a staff member is in charge of discussing PA events with local policy stakeholders and verifying that each planned event is: *(i)* genuinely a PA event (and not just a ‘cultural’ event such as a public performance), *(ii)* takes place at weekend during the volitional phase of the GLMC (i.e.*,* between mid-March and mid-April, see Fig. 1), *(iii)* is suitable for families to participate in together and *(iv)* free of charge for families.

### Step 2: the GLMC for children and parents

**Fig. 4:**
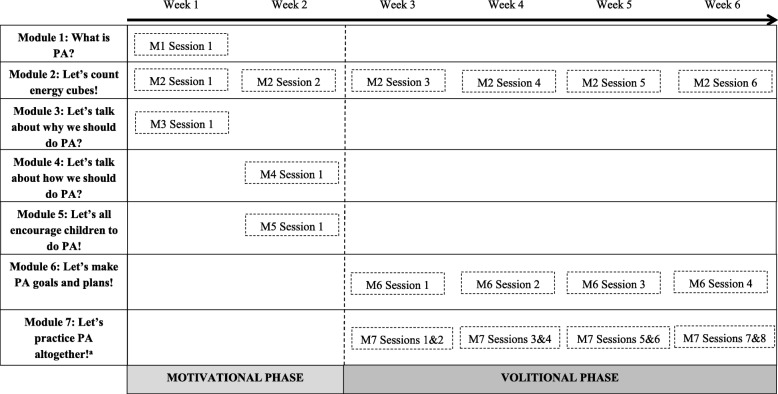
General scheme of the great live and move challenge intervention. *Note.* ^a^ Sessions in the module 7 are concrete PA sessions (i.e., “PA events”) (see Table 1)

As displayed in Fig. 4, the GLMC intervention consists of 7 different modules of PA promotion (22 sessions in total) spread over 6 weeks. Some modules consist of a single session delivered during a specific week of the intervention (e.g.*,* Module 3: ‘Let’s talk about why we should do PA’; week 1), whereas others consist of several sessions, one session being repeated over several weeks (e.g.*,* Module 6: ‘Let’s make PA goals and plans!’, one session is repeated 4 times over weeks 3 to 6). Each module of the GLMC uses a specific behavioral change technique (BCT) to target a TPB variable; the choice of BCT is based on current recommendations for theory-based interventions [45–47]. A detailed description of the modules and session content is given in Table 1.

**Table 1:** Presentation of the modules of the Great Live and Move Challenge intervention

Module	No. of sessions	Main BCTs used	Main targeted psycho-social variables	Scientific evidence [ref]	Description of the sessions content	Implemented by
Module 1: What is PA?^a^	1 session	Provide information on consequences of behavior in general	Subjective norms	[70]	M1 Session 1 (Week 1 – duration: 45 min) • *Presentation of the notion of PA:* definition of PA, the different ways of doing PA (i.e.*,* sport activities, leisure time PA, lifestyle PA). • *Presentation of the PA recommendations for children:* 60 min of moderate to vigorous PA per day [5]. • *Broadcasting of the video entitled “Presentation of the GLMC”:* Presentation of the general principles of the GLMC e.g.*,* the importance to practice PA with friends and family members, the absence of any competitive aspect, and the GLMC schedule.	• Teachers
Module 2: Let’s count energy cubes!^a^	6 sessions	Prompt self-monitoring of behavior	Perceived behavioral control, shared family PA	[71]	M2 Session 1 (Week 1 – duration: 30 min) • *Distribution and presentation of the GLMC diary for children.* • *Presentation of the notion of “energy cube”:* one “energy cube” is equivalent to 15 min of continuous PA, when practicing family PA the child cumulates additional “energy cube(s)” for each family member who has practiced with him/her. M2 Session 2 (Week 2 – duration: 30 min) • *Learning to count ‘energy cubes’:* exercises to learn how to correctly count the number of ‘energy cubes’ when practicing PA with and without family members, exercises to learn how to correctly complete the table of counting of ‘energy cubes’ in the GLMC diary. M2 Session 3 (Week 3 – duration: 15 min) • *Registering ‘energy cubes’:* Taking stock of the number of ‘energy cubes’ cumulated by children, assistance for children who encounter difficulties to count their ‘energy cubes’. M2 Session 4 (Week 4 – duration: 15 min) • *Registering ‘energy cubes’:* cf. M2 session 3. M2 Session 5 (Week 5 – duration: 15 min) • *Registering ‘energy cubes’:* cf. M2 session 3. M2 Session 6 (Week 6 – duration: 15 min) • *Registering ‘energy cubes’:* cf. M2 session 3.	• Teachers
Module 3: Let’s talk about *why* we should do PA?^a^	1 session	Provide information on consequences of behavior to the individual	Attitudes	[72]	M3 Session 1 (Week 1 – duration: 45 min) • *Broadcasting of the video no. 1 entitled “Let’s talk about PA – The advantages to regularly practice PA”:* Children have to guess the advantages to regularly practice PA presented in the film (i.e.*,* “to be in a good mood”, “to have fun”, “to improve one’s skills”, “to prevent injuries”, “to have a great time with family and friends”, “to be in a good health”, “to stay focused at school”). • *Debate:* children discuss about their perceived personal advantages to practice PA.	• Teachers
Module 4: Let’s talk about *how* we should do PA?^a^	1 session	Provide information on where and when to perform the behavior	Perceived behavioral control	[73]	M4 Session 1 (Week 2 – duration: 45 min) • *PA collective repertory:* Elaborating a collective repertory for each class about the PA that a child could practice as well as where, when and with who practicing those activities. • *Broadcasting of the video no. 2 entitled “Let’s talk about PA – How to regularly practice PA”:* tips about the activities, the locations, the persons and the moments of the week a child can practice PA. • *Linking exercise:* Identification for each child of some realistic combinations linking a new PA to do, where, when and with whom practicing those activities.	• Teachers
Module 5: Let’s all encourage children to do PA! ^b^	1 session	Provide information on consequences of behavior to the individual	Parental involvement in shared family PA, parental PA social support	[74]	M5 Session 1 (Week 2 – duration: 45 min) • *Presentation of the Powerpoint slides entitled “how to promote PA in our children?”:* Presentation of the benefits of shared family PA (i.e.*,* “to have fun”, “to strengthen family relationships”, “to lower the risk of disease for all the family members”); and presentation of the benefits of supporting ones’ child toward PA (i.e.*,* “helping children to find flourishing activities”, “helping children to take responsibility for their health”). • *Creation of “PA events” for children:* Discussion between all the actors of the educative community (e.g.*,* teachers, parents, local policy stakeholders) about how creating “PA events” altogether during the GLMC.	• Teachers • Municipal officials • Local policy stakeholders
Module 6: Let’s make PA goals and plans!^a^	4 sessions	• Action planning • Goal setting	• Planning • Intentions	[75]	M6 Session 1 (Week 3 – duration: 15 min) • *Planning:* Each child plan the number of ‘energy cubes’ he/she will try to cumulate during the coming week in different areas of life (i.e.*,* recesses, lunch times) • *Goal setting*: Each child set a goal of total ‘energy cubes’ to cumulate during the coming week. M6 Session 2 (Week 4 – duration: 15 min) • *Planning:* cf. M6 Session 1 • *Goal setting*: cf. M6 Session 1 M6 Session 3 (Week 5 – duration: 15 min) • *Planning:* cf. M6 Session 1 • *Goal setting*: cf. M6 Session 1 M6 Session 4 (Week 6 – duration: 15 min) • *Planning:* cf. M6 Session 1 • *Goal setting*: cf. M6 Session 1	• Teachers
Module 7: Let’s practice PA altogether! ^a,b^	8 sessions	Implementation of PA sessions^c^	Perceptions of activity opportunities	[46]	M7 Sessions 1 & 2 (Week 3 – duration: variable, depending on “PA events”) • *Session 1, a “PA event” in the schools:* Teachers of each school organize a “PA event” for children during school hours (e.g.*,* Olympiad, treasure hunt, team based sport activities). • *Session 2, a “PA event” in the city:* Local policy stakeholders organize a “PA event” designed for families during the week-end in one quarter of the city (e.g.*,* family hike, giant Zumba lesson). M7 Sessions 3 & 4 (Week 4 – duration: variable in function of the ‘PA events’) • *Session 3, a “PA event” in the schools:* cf. M7 Session 1. • *Session 4, a “PA event” in the city:* cf. M7 session 2. M7 Sessions 5 & 6 (Week 5 – duration: variable in function of the ‘PA events’) • *Session 5, a “PA event” in the schools:* cf. M7 Session 1. • *Session 6, a “PA event” in the city:* cf. M7 session 2. M7 Sessions 7 & 8 (Week 6 – duration: variable in function of the ‘PA events’) • *Session 7, a “PA event” in the schools:* cf. M7 Session 1. • *Session 8, a “PA event” in the city:* cf. M7 session 2.	• Teachers • Municipal officials • Local policy stakeholders

*Note*. *BCT* behavior change technique, *GLMC* great live and move challenge, *PA* physical activity, *TPB* theory of planned behavior, *M* Module

^a^session indented for children; ^b^ session indented for parents; ^c^ such a behavior change technique is not referenced in the taxonomy of Michie and collaborators [45]

The intervention includes the viewing of 3 videos specifically produced for the GLMC. The first video is called “Presentation of the GLMC” (M1 Session 1) and aims to present the GLMC and federate participants around the program. The second and the third videos are called “Let’s talk about PA – The advantages to regularly practice PA” (M3 Session 1) and “Let’s talk about PA – How to regularly practice PA” (M4 Session 1), respectively. In accordance with the principles of the GLMC intervention, those 2 videos are theoretically based, that is, they have been designed and produced to specifically have an impact on some TPB variables. More precisely, based on the existing literature concerning salient beliefs among children (e.g.*,* [72]), the video included in the module 3and entitled “Let’s talk about PA – The advantages to regularly practice PA” targets affective and instrumental attitudes of the children toward PA (e.g.*,* ‘to have fun’, ‘to be in a good health’). The video entitled “Let’s talk about PA – How to regularly practice PA”, included in the module 4, targets the perception of behavioral control of children through the provision of advices by children, parents, elite athletes and doctors on what activities, when, where and how to practice PA

### Step 2a: the motivational phase

The motivational phase targets the TPB determinants of intentions (i.e.*,* attitudes, subjective norms and perceived behavioral control). Module 1 is entitled ‘What is PA?’ and targets subjective PA norms using the BCT called ‘Provide information on consequences of behavior in general’ [45]. This module consists mainly of presenting the recommendations for children’s PA (i.e.*,* 60 min of moderate to vigorous PA per day) [4, 5]. Module 2 is called ‘Let’s count energy cubes!’ and targets perceived behavioral control and shared family PA through the use of the ‘prompt self-monitoring of behavior’ BCT. The GLMC uses an adapted form of the self-monitoring technique designed to be fun for children to use. Children quantify their PA level by accumulating ‘energy cubes’. An energy cube is equivalent to 15 min of continuous PA. The ‘energy cube’ concept was inspired by the ‘Great Pierre Lavoie Challenge’, an intervention that has been implemented in more than 50% of schools in Quebec Province in Canada since 2009. The energy cube was chosen as the unit of measurement because it is a clear, simple, feasible and culturally adapted symbol for promoting PA amongst children (https://www.legdpl.com). In order to promote family PA in the GLMC, a child also receives ‘energy cubes’ for family members’ PA when s/he performs any kind of PA with a family member for at least 15 min. For example, if a girl taking part in the GLMC goes for a 15-min bicycle ride with her mother and her brother she accumulates 3 ‘energy cubes’. Module 2 is the only module common to the motivational and volitional phases (see Fig. 4). During the motivational phase of the GLMC, module 2 session (i.e.*,* M2 sessions 1 and 2) primarily consist of teaching children how to count ‘energy’ cubes and record them in their GLMC diary (see Table 1). Module 3, called ‘Let’s talk about why we should do PA’, targets attitudes through the use of the ‘provide information on consequences of behavior to the individual BCT. In this module children watch a movie presenting some of the advantages of regular PA (*e.g.,* ‘having fun’) and then discuss how they think they would benefit from undertaking PA. Module 4, called ‘Let’s talk about how we can do PA’ targets perceived behavioral control through the use of the ‘provide information on where and when to perform the behavior’ BCT. First, children watch a movie which gives information about different kinds of PA and tips about where, when and with whom to do PA. Then each child thinks about a new form of PA and when, where and with whom s/he will do this new PA. Module 5 is called ‘Let’s all encourage children to do PA!’ and is delivered to parents. This module targets parental support for children’s PA and parental involvement in family PA (see Fig. 3). The BCT named ‘provide information on consequences of behavior to the individual’ is used in this session to promote parental involvement. During a 30-min round-table discussion parents are informed about the benefits of shared family PA (e.g.*,* ‘strengthens family relationships’) and the benefits of supporting their children’s PA (e.g.*,* ‘helps children to take responsibility for their health’).

### Step 2b: the volitional phase

The volitional phase aims to help children to translate their intentions into good PA habits and so the sessions in this phase target the most proximal determinants of PA in the model underlying the GLMC (i.e.*,* perceived behavioral control, intentions, planning and perceptions of activity opportunities) (see Fig. 3). Modules 2, 6 and 7 are included in this phase, and the sessions comprising these modules are repeated every week over weeks 3 to 6.

During the volitional phase, module 2 consists of inviting children to record their PA every day, by counting the ‘energy cubes’ they have accumulated and writing this down in their GLMC diary. During the volitional phase module 2 sessions (i.e., M2 sessions 3, 4, 5, 6; see Table 1) teachers record the total number of ‘energy cubes’ accumulated by the children in their class every week. Module 6 is called ‘Let’s make PA goals and plans!’ and targets both planning and intentions variables in children, through the use of ‘action planning’ and ‘goal setting’ BCTs respectively. During each week of the volitional phase, children are asked to plan the number of ‘energy cubes’ they will aim to cumulate for the week in different areas of life (i.e.*,* recesses, lunch times). They are then asked to set a realistic goal of “energy cubes” they will try to cumulate. Finally, Module 7, called ‘Let’s do PA together!’ directly targets perceptions of activity opportunities (note that this component is not included in Michie’s BCT taxonomy [45]). As shown in Fig. 4, children have access to at least two PA events in every week of the volitional phase: *(i)* one organized by their teachers that takes place during school hours (e.g.*,* Olympiad), and *(ii)* one weekend activity designed for families and organized by local policy stakeholders in each community of communes (e.g.*,* giant zumba). There may also be a weekly PA event delivered by municipal officials in recreation centers These PA events will be promoted through advertisement in local papers, posters in streets and schools and the French website of the GLMC (http://www.gdvb.fr).

### Evaluation

All variables will be measured at four time points (Fig. 1) i.e.*,* pre- and post-intervention in each year of the two-year intervention:

- year 1: pre-intervention 1 (T0); baseline;
- year 1: post-intervention 1 (T1); at the end of intervention 1, 4.5 months after baseline;
- year 2: pre-intervention 2 (T2);12 months after baseline, 7.5 months after the end of intervention 1;
- year 2: post-intervention 2 (T3); end of intervention 2, 16.5 months after baseline.

#### Evaluation of children

##### Self-reported PA

PA is measured using an adapted version of the self-administered Physical Activity Questionnaire for Children (PAQ-C) [48] which covers PA undertaken in *(i)* sporting clubs or associations, *(ii)* break times at school, *(iii)* lunch times, *(iv)* free time and *(v)* as part of daily life. Children are asked to report the specific names of activities in which they have engaged together with the frequency and duration of each activity over the last 7 days. Total PA and PA in each domain are then calculated in hours per week. The validity and reliability of the PAQ-C have been demonstrated [49].

##### Objectively measured PA

Triaxial Actigraph GT3X+ accelerometers (Actigraph; Pensacola, FL) will be used to measure PA in a subsample of 400 children. As a triaxial accelerometer, the GT3X captures movement data in three orthogonal directions (vertical, forward-backward and lateral). In line with current recommendations, children will be asked to wear the accelerometer around their waist for at least four consecutive days [50]. The validity of the Actigraph GT3X+ accelerometer as a method of estimating children’s PA has already been assessed [51].

##### Psychosocial variables

The psychosocial variables to be measured are the TPB variables (i.e.*,* attitudes, subjective norms, perceived behavioral control and intentions) and the variables added to the theoretical model underlying the GLMC to fill the intentions-behavior gap (i.e.*,* planning, perception of activity opportunities) (Fig. 3). All 30 questionnaire items have been tested in feasibility studies (details of feasibility testing are given below). Responses to all items are given using a four-point scale: 1 (strongly disagree); 2 (disagree); 3 (agree); 4 (strongly agree).

##### TPB variables

The TPB questionnaire is based on current guidelines [52] and previous TPB research amongst children [29, 31, 32, 53]. Intentions are measured using two items (e.g.*,* ‘Do you intend to engage in PA almost every day of this week?’). Attitudes are measured using six items: three dedicated to the affective component (e.g.*,* ‘Is engaging in PA almost every day fun for you?’) and three to the instrumental component (e.g.*,* ‘Does engaging in PA almost every day improve your physical health?’). Subjective norms are measured using eight items: four items assess the injunctive component (e.g.*,* ‘Would your friends like you to do PA almost every day?’), and four measure the descriptive component (e.g.*,* ‘Does your father engage in PA almost every day?’). Perceived behavioral control is assessed with four items (e.g.*,* ‘Do you think you can do PA almost every day even if you have homework to do?’).

##### Planning and activity opportunities variables

Planning will be measured with five items based on the French version of the Action Planning Scale [54] (e.g.*,* ‘Do you know when you will be engaged in PA during the next week (*e.g., times* of day, day(s) of the week)?’) and perceptions of activity opportunities was measured using five items based on previous studies [29, 43, 55] (e.g.*,* ‘Aside from physical education lessons, do you have opportunities for PA at school, in the playground or in a sports hall?’).

#### Evaluation of parents

##### Shared family PA

Parents will be asked to report their commitment to shared family PA using a questionnaire developed by Rhodes et al. [56] which asks about the frequency and duration of structured and unstructured activities they engage in as a family (i.e.*,* with at least one child) in a typical week. Examples of structured (e.g.*,* parent–child swimming lessons) and unstructured (e.g.*,* family walks) activities are given to clarify these terms. The total duration of parental participation in family PA will then be calculated in hours per week.

##### PA social support

Parental support for children’s PA will be measured with a three-item self-administered questionnaire inspired by the ‘praise and understanding’ subscale of the Parental Involvement In Sports Questionnaire [57] (e.g.*,* ‘I regularly praise my child for all his/her PA’). Responses to items are given using a seven-point scale ranging from (1) strongly disagree to (7) strongly agree.

##### Feasibility studies

Two preliminary pilot studies were conducted to confirm that it is feasible to deliver the GLMC intervention and evaluate the participating children and parents. These studies also represented an opportunity to assess the strengths and weaknesses of the intervention.

The first pilot study was carried out in and involved 306 children aged 7 to 11 years old attending 6 public schools and 104 of their parents. During this study we noted that children found the concept of ‘energy cubes’ very attractive; in fact they became something of a fad. We also found that the format of parents’ meetings (see Module 5, Table 1) were more attractive when they were held at the children’s schools rather than in town halls and organized as ‘round-table discussions about promoting PA’ with teachers, municipal officials and local policy stakeholders rather than organized as ‘PA promotion presentations’.

In 2015 a second study involving 793 children aged 7 to 11 years old from 16 public schools and 329 of their parents was carried out. This pilot study was used to test the feasibility of the evaluation procedure and the potential for recruitment. We found that it was feasible to administer the children’s questionnaires (evaluating self-reported PA, TPB variables and additional psychosocial variables) in a classroom setting (i.e.*,* 50–55 min for the completion of the questionnaire for a class of 20–25 children). We also confirmed the feasibility of the procedure for distributing and retrieving accelerometers from a subsample of children. The parents’ questionnaire was also reported to be feasible. Sixteen of the 53 schools (30%) that were contacted agreed to participate and the rates of return of signed consent forms were 86.7% for the child’s participation in the study, 37% for the wearing of an accelerometer by the child and 33.6% for completion of the parents’ questionnaire.

### Statistical considerations

#### Power analysis

Without considering the clustering effect, it is calculated that 180 children per randomization group are needed to observe a 15% difference in the proportions of children in the intervention and control groups achieving the current internationally recommended level of PA with 80% power, given a two-tailed type I error rate of 5%. We assumed that 35% of children would be achieving the current internationally recommended PA level of 60 min of moderate to vigorous PA per day at baseline.

The clustering structure of the randomization requires a variation inflation factor equal to *1 + (m(1 + cv*^*2*^*)-1)r* [58] where *m* is the mean number of children per cluster; *cv* is a coefficient of variation equal to ratio of the standard deviation of the cluster sizes and *m*; *r* is the intraclass correlation coefficient (ICC) for the communes of one community of communes (i.e.*,* one cluster) and reflects the degree of cluster homogeneity. *r* was set at 0.005 based on preliminary data obtained from pilot studies and the database of ICCs [56] .

Given the number of children in selected community of communes clusters (*m* = 781 children on average; *cv* = 1.05), 3400 subjects are required to observe a 15% difference in the proportion of each group reaching the current international recommendation of 60 min with 80% power. Assuming a loss-to-follow-up rate of 15%, a reasonable estimate of the required sample size is 4000 subjects, i.e.*,* 2000 in the intervention group and 2000 in the control group, corresponding to around 160 classes.

#### Statistical analysis

Data will be analyzed on an intention-to-treat basis using Stata software (version 13, StataCorp, College Station, USA). All the statistical tests will be two-sided and *p*s < 0.05 will be considered significant. The number of included subjects and the enrolment curve, the number of theoretical follow-ups corresponding to the number of included subjects, the number of follow-ups actually carried out and the relation between the two will be presented by the randomization group. The cumulated follow-up duration will be calculated and the actual cumulated follow-up duration expected will be presented. The data analysis, carried out on intention to treat will include: (a) the comparison of the enrolment groups (for both the children and the children characteristics), (b) the analyses described below, (c) the missing data sensitivity analysis to determine the statistical nature of the longitudinal data (Missing At Random or Not Missing At Random).

##### Data entry and data management

In the GLMC trial, all data will be electronically processed. Original study forms will be entered and kept on file at the participating site. Participant files are to be stored in numerical order and stored in a secure and accessible place. Participant files will be maintained in storage for a period of 10 years after completion of the study.

Additional errors will be detected by programs designed to detect missing data or specific errors in the data. The Data Manager will respond by checking the original forms for inconsistency, checking other sources to determine the correction, modifying the original (paper) form entering a response to the query.

##### Effectiveness analysis

Baseline similarity of the intervention and control groups will be assessed at cluster level (i.e.*,* communities of communes) and child level using descriptive statistics and *T*-tests or Mann-Whitney tests for continuous variables and χ^2^ or Fisher’s exact tests for categorical variables.

Linear mixed models will be used to determine the impact of the intervention impact; this will allow us to calculate fixed effects, such as the effect of randomization group, and random effects such as the effect of subjects and clusters given the potential for intraclass correlations (between and within subjects and clusters). Longitudinal analysis of the dichotomous primary endpoint (i.e.*,* achieving or not achieving the current internationally recommended PA level of 60 min of moderate to vigorous PA per day) will be conducted with a logit link function, effects of subject, group, time and cluster and the time*group interaction will be calculated. The effect of the intervention will be evaluated at child (and parent) level, but also at school, commune and community of communes levels. Potential confounders such as age and sex will be included in the model as covariates.

The same analytical approach will be applied to the secondary outcome variables. The effect of the intervention on TPB variables and other psychosocial continuous variables will be assessed using similar linear mixed models, with a regress link function.

##### Analysis of the mediating effect of psychosocial variables

Mediation analyses are an important step in evaluation of theory-based interventions as they help to determine the extent to which behavioral change during the intervention is explained by changes in the variables included in the underlying theoretical model [24]. To test whether the effect of the GLMC on children’s PA levels is mediated by the variables specified in the underlying theoretical model (Fig. 3) we will perform sequential mediation analyses using the SPSS (version 21) macro PROCESS, with 10,000 bootstrapped replications [59]. PROCESS allows one to conduct multiple mediator analysis in linear multiple regression models whilst accounting for covariates (e.g.*,* age, sex, PA level at T0). The independent variable of the model will be the group, (coded 1 for the intervention group and 0 for the control group). The dependent variable will be PA level at T3. The multiple mediation analyses will include 3 sequences of mediators (see [60, 61] for a similar approach). Change in parental shared family PA and parental social support for PA between T0 and T3 will constitute the first sequence of mediators, change in children’s attitudes, subjective norms and perceived behavioral control between T0 and T3 will constitute the second sequence of mediators, and change in children’s intentions, perception of activity opportunities and planning between T0 and T3 will constitute the third sequence of mediators. In addition, moderation analyses will be conducted to test whether the effects of change in intentions on the level of PA of children is moderated by different levels of perceptions of activity opportunities and different levels of planning (see Fig. 3). The results of analyses will be presented using two types of coefficients: a regression coefficient (*β*) for each parameter and an indirect effect coefficient (*θ*) for each indirect pathway (via a specific mediator) between the independent variable (group) and the dependent variable (PA at T3). All the statistical tests will be two-sided and *p*s < 0.05 will be considered significant.

## Discussion

The GMLC study takes place in the context of high levels of physical inactivity in children across the world [4, 6, 11]. The prevalence of inadequate levels of PA has reached 69% in France [7] and the GLMC study aims to increase the proportion of children achieving the World Health Organization’s recommended level of PA (60 min of moderate to vigorous PA per day) by at least 15%. The GLMC study is a two-year cluster-randomized controlled trial in French primary school children aged 7–11 years and the design has several strengths: it involves an integrated multilevel PA promotion intervention targeting schools, families and their communities simultaneously; the GLMC intervention is theory-based, on an extended version of the TPB; the study will involve comprehensive evaluation of objective ad self-report PA data and psychosocial variables in children and their parents, at four different times over a two-year period.

Although there are numerous interventions designed to promote PA in young people [12, 13], the effects on children’s PA have generally been very small (*d* < 0.10) [12] and the lack of high quality evaluations makes it difficult to draw firm conclusions about their effectiveness, especially in children [18]. Furthermore little is known about the psychosocial factors which influence the behavioral change produced by these interventions [18]. Thanks to the originality of its design, its theoretical foundations and the combination of objective and self-reported evaluations of PA, the GLMC study is expected to add to knowledge about the effectiveness of a multi-component intervention designed to promote PA promotion amongst children and the psychosocial mechanisms that underlie its impact.

The design of the GLMC intervention is consistent with an ecological approach to promotion of health behaviors [14]. With a three-level integration (i.e.*,* school, family, and community involvement), the GLMC intervention is explicitly based on the involvement of a large community of education stakeholders (i.e.*,* parents, teachers, municipal officials and public policy stakeholders). The aim of this approach is to ensure that children receive the relevant behavioral prompts (e.g.*,* to be more physically active) from a variety of sources (e.g.*,* parents; teachers; municipal officials) and in a variety of settings (e.g.*,* home; school; community) [14]. Partnerships between education stakeholders appear to be one of the key factors in the efficacy and sustainability of educational interventions [62, 63], hence one of the ambitions of the GLMC is to encourage dialogue and exchange of information regarding PA promotion between several strata of education stakeholders. In fact, this is the main goal of module 5 of the GLMC (Table 1), which is intended to provide an opportunity for parents, teachers, municipal officials and local policy stakeholders to collaborate more closely to improve children’s health.

The GLMC intervention is a short but intense intervention, meaning it is easily repeatable. The GLMC intervention can be implemented annually over a six-week period during which 21 children’s sessions are delivered, a mean of 3.5 sessions per week. The short duration and high intensity were chosen for several reasons. First, most TPB-based interventions are quite brief (i.e.*,* lasting a few days or weeks) [64] as they target some particularly modifiable behavior-related cognitions (i.e.*,* attitudes, subjective norms, perceived behavioral control) [65]. Second, six weeks appears to be sufficient to allow new patterns of PA to become habitual in most individuals [66]. Third, the GLMC was explicitly designed to create an intense focus on PA promotion for a short period every year in a particular educational community. The annual repetition of this period of intense PA promotion should help to maintain new PA habits developed during a child or family’s first year of participation in the GLMC. It is worth noting that most previous reports on children’s PA interventions have not clearly reported the duration or intensity of the intervention, making difficult to evaluate how these factors moderate the efficacy of such interventions [12]. This cluster-randomized trial will provide information about the effectiveness of a short and intense multi-component PA promotion intervention delivered annually for two years.

Another potential key contribution of the GLMC is related to its strong theoretical foundation. In line with current recommendations [24, 45–47] each component of the intervention has been designed specifically to affect psychosocial variables and ultimately to promote behavioral change in accordance with a specific theoretical model. The theoretical foundations of the GLMC intervention influenced the content of the children’s sessions and are also communicated to the various local partners involved (i.e.*,* parents, teachers, municipal officials and local policy stakeholders) according to their specific role (i.e.*,* they are sensitized to the variables and perceptions on which they can have a key impact). Educational stakeholders have been reported to be particularly inclined to base their health interventions on psychosocial theories [67]. Pedagogical and practical guides have been created to explain the theoretical approach underlying the GMLC to specific groups of educational stakeholders, as it has been reported that such guides are one of the keys to the efficacy of behavioral theory-based public health interventions [68]. The GLMC study will also address some of the issues and research questions related to behavioral theories that underlie PA interventions [69]. We propose to test a model of behavioral change based on an extended version of the TPB, by prospectively measuring the TPB variables and two additional variables, PA planning and perceptions of activity opportunities, and using them to explain children’s PA behavior. As there is an intentions-behavior gap under the current version of the TPB model [37], we will use mediation and moderation analyses to determine empirically the impact of the GLMC on PA planning and perceptions of activity opportunities for activity, which may positively moderate the intentions–PA behavior relationship (Fig. 3). The results of these analyses should contribute to debate on refinement of the TPB model [41].

The GLMC study will evaluate the effectiveness of a multilevel, TPB-based primary school PA program and will potentially provide valuable information for schools and public health policy officers looking for innovative PA programs. One of the intended long-term, indirect final outcomes of the GLMC program is to prevent the development of metabolic and non-communicable diseases (e.g.*,* obesity, diabetes, cancer) in all family members exposed to the intervention, by promoting PA to children and their parents in association with the educational community. We believe that the GLMC intervention has a great potential to be welcomed by the general population as an enjoyable project that promotes health and social collaboration.

## Abbreviations

BCT: Behavioral change technique
GLMC: Great Live & Move Challenge
MCI: Montpellier cancer institute
PA: Physical activity
TPB: Theory of planned behavior
